# *eDRAM*: Effective early disease risk assessment with matrix factorization on a large-scale medical database: A case study on rheumatoid arthritis

**DOI:** 10.1371/journal.pone.0207579

**Published:** 2018-11-26

**Authors:** Chu-Yu Chin, Sun-Yuan Hsieh, Vincent S. Tseng

**Affiliations:** 1 Computer Science and Information Engineering, National Cheng Kung University, Tainan, Taiwan; 2 Computer Science and Information Engineering, National Chiao Tung University, Hsinchu, Taiwan; Universitatsmedizin Greifswald, GERMANY

## Abstract

Recently, a number of analytical approaches for probing medical databases have been developed to assist in disease risk assessment and to determine the association of a clinical condition with others, so that better and intelligent healthcare can be provided. The early assessment of disease risk is an emerging topic in medical informatics. If diseases are detected at an early stage, prognosis can be improved and medical resources can be used more efficiently. For example, if rheumatoid arthritis (RA) is detected at an early stage, appropriate medications can be used to prevent bone deterioration. In early disease risk assessment, finding important risk factors from large-scale medical databases and performing individual disease risk assessment have been challenging tasks. A number of recent studies have considered risk factor analysis approaches, such as association rule mining, sequential rule mining, regression, and expert advice. In this study, to improve disease risk assessment, machine learning and matrix factorization techniques were integrated to discover important and implicit risk factors. A novel framework is proposed that can effectively assess early disease risks, and RA is used as a case study. This framework comprises three main stages: *data preprocessing*, *risk factor optimization*, and *early disease risk assessment*. This is the first study integrating matrix factorization and machine learning for disease risk assessment that is applied to a nation-wide and longitudinal medical diagnostic database. In the experimental evaluations, a cohort established from a large-scale medical database was used that included 1007 RA-diagnosed patients and 921,192 control patients examined over a nine-year follow-up period (2000–2008). The evaluation results demonstrate that the proposed approach is more efficient and stable for disease risk assessment than state-of-the-art methods.

## Introduction

Rheumatoid arthritis (RA), a systemic autoimmune rheumatism disease (SARD), is rare and causes chronic bone damage and deterioration. RA is difficult to diagnose at an early stage, and with disease progression, RA may lead to bone deformation, swelling, pain, and permanent disability [1, 2]. Unfortunately, this disease is not easily cured and requires long-term follow-up, controller medications, and regular healthcare visits. Although RA does not directly cause death, it can clearly reduce the patient’s ability to work or live independently, as it affects a wide range of activities, such as walking, eating, personal hygiene, and even mental health [1, 3, 4]. This significantly increases long-term domestic expenditure and affects national productivity and medical resource allocation [5, 6]. Accordingly, early detection of RA has been extensively studied [7–16] over the past few years, as it allows effective symptom management and prevents joint deterioration by appropriate medication therapy. Therefore, early diagnosis of this serious disease is fundamental in a successful treatment strategy [1, 12–14, 17]. Thus, disease prediction for RA is an important issue in medical informatics.

Recent advances in electronic medical record (EMR) standardization and medical information exchange systems have substantially enlarged EMR data sets. Efficient and effective analytical techniques are important for discovering new medical knowledge from big EMR data. The discovered rules can be used to improve disease prediction and prevention, assess patient prognosis, and increase diagnostic precision.

There are two issues in EMR analysis. First, a small number of diagnostic records are inadequate to represent the complete picture of a patient’s health status. For instance, symptoms of several serious diseases, such as cancer, are obscure during early disease development stages. Therefore, the lack of patient medical records may lead to misdiagnosis, resulting in delayed medical treatment and proper care. Secondly, personal EMRs are scattered in a number of hospitals because patients are not confined to one hospital for treatment. Thus, it is difficult to combine personal EMR data for analysis, and the possibility of misdiagnosis increases.

To address these issues, a universal National Health Insurance (NHI) program was conducted in Taiwan to generate a database called *NHIRD* (*National Health Insurance Research Database*) containing physician diagnostic records and prescriptions. Large-scale medical information is recorded by physician visits; this information is diverse and has been collected from all hospitals in Taiwan. Moreover, it is suitable for investigating personal health trends. NHIRD has great potential for discovering novel information, such as hidden disease risk factors, the causal relationship between diseases and symptoms, disease development, and a disease risk assessment model to promote treatment.

**Fig 1:**
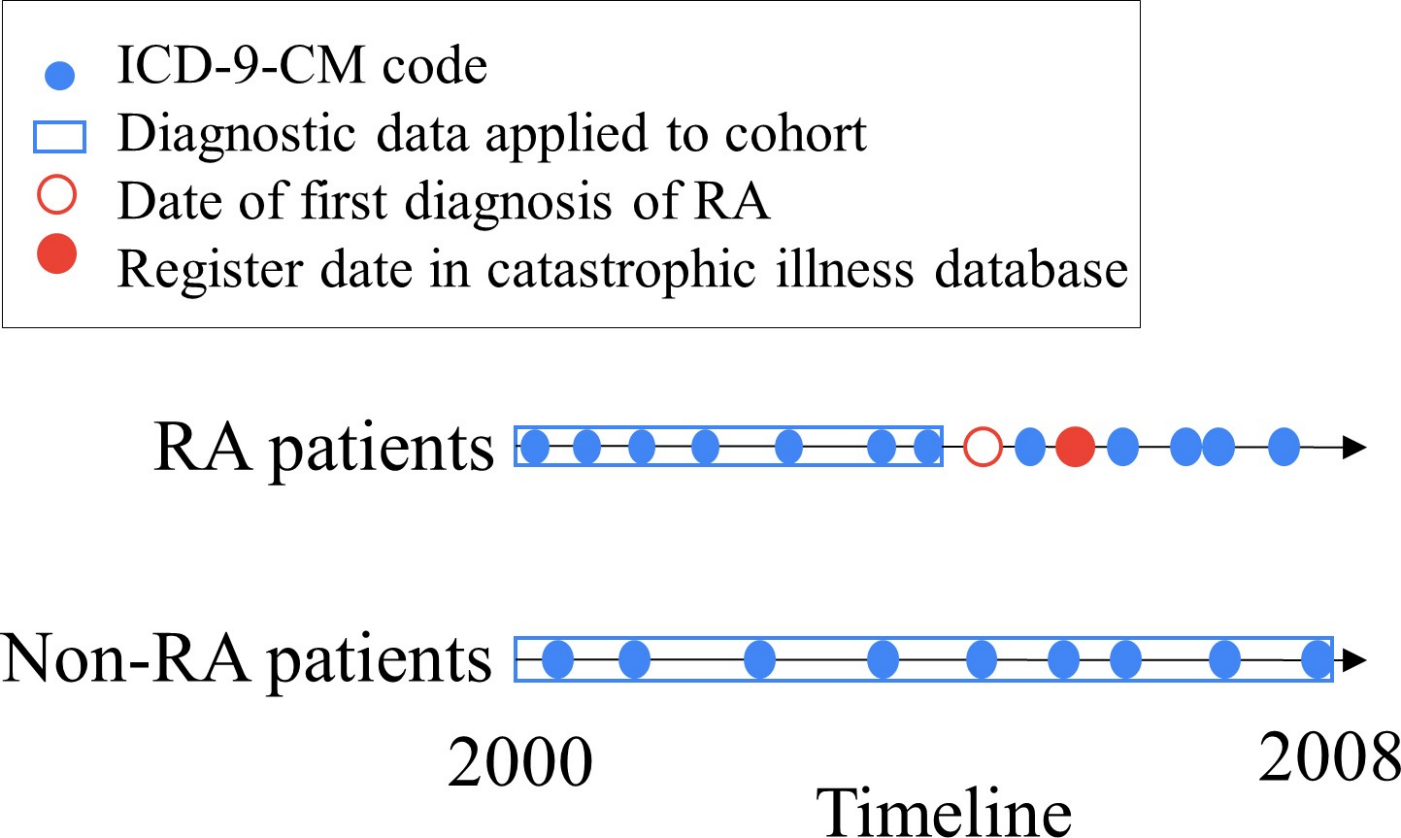
Timeline for data collection and definition of RA patients.

Although a number of past studies have considered this issue [8, 11, 18–25], it is difficult to design a disease risk assessment system that can accurately reflect the health status of a patient. This has recently attracted attention owing to the need for improving the accuracy of disease prediction, based on information in large-scale EMR databases. Thus, analytical techniques have been proposed. Liao, *et al*. (2011) developed classification algorithms by using penalized logistic regression. To validate the proposed algorithm, it was applied to two external hospitals using different electronic health record (EHR) systems [8, 11]. Carroll, *et al*. (2011) applied support vector machines (SVMs) to identify RA cases using EHR (ICD-9 codes, medications, and natural language processing-derived clinical notes) [9]. Kuo, *et al*. (2013) utilized SVMs to predict the onset of bullous pemphigoid, and the risk factors were determined by logistic regression [18]. Rau, *et al*. (2015) used artificial neural networks and logistic regression to construct a prediction model for liver cancer development in patients with type II diabetes mellitus. Furthermore, a user interface was designed to compute the probability of liver cancer occurrence using physician-proposed risk factors [19]. Chin, *et al*. (2015) proposed a framework based on associative classification for mining risk patterns to assess the onset of early RA, and the mined classifiable patterns exhibited highly significant associations with disease risk. To estimate the novelty of risk patterns, a method for calculating the number of related studies integrated in the PubMed search engine was included in the analysis stage of the framework [26]. These associative classification techniques are based on frequent and high confidence association rules to classify objects. Classification based on multiple association rules (CMAR) [27] and classification based on associations [28] are effective associative classification techniques. Cheng, *et al*. (2017) proposed a framework integrating the “classify-by-sequence” (CBS) method [29] to mine for sequential risk patterns from time-series information in diagnostic records for early assessment of chronic diseases [30]. CBS and BayesFM [31] are sequential classification techniques that primarily combine the algorithms of sequential pattern mining, rule selection, and classification. In the above studies, the classifiable sequential patterns and classifiable patterns are considered disease risk factors for evaluating disease progression. Patient phenotyping is used to identify patients who match criteria from a large-scale population. The features of EHR are utilized to identify the cohort by machine learning and statistical methods [25]. In this framework, early disease risk assessment is aimed at discovering hidden factors and establishing assessment models based on diagnostic data that are collected before formal diagnosis of the target disease, such as RA (Fig 1). By using the model, the target disease can be assessed before its onset. This is generally called early disease risk assessment [30].

Non-negative matrix factorization (NMF) is an unsupervised analytical technique for parts-based representation that achieves significant reduction in the dimensions of objective data and discovers latent factors [32–40]. It has been successfully applied to image recognition and text mining and has effectively improved accuracy and efficiency [33]. Recently, various analytical methods have been developed for different types of medical data by using the NMF algorithm. For example, Yang, *et al*. (2016) proposed unsupervised clustering to analyze gene expression data [36], Paine, *et al*. (2016) proposed unsupervised analysis using desorption electrospray ionization datasets [39], and Ozaki, *et al*. (2016) proposed analysis of complex actions in sports from electromyography data [41]. However, the above studies neglected the investigation of diagnostic data. Moreover, they have several limitations: 1) Identifying risk factors requires expert advice [42, 43]. For a large amount of medical data, the trend is to identify risk factors without human supervision. 2) A large data size or number of risk factors requires longer execution time and results in lower assessment accuracy. However, in medical decision-making, both efficiency and assessment accuracy should be considered. Thus, the ability of NMF to significantly reduce dimensionality and maintain data quality is important. 3) Recent studies have shown that SVM is useful for identifying phenotyping [9, 11, 18, 44]; however, an overly large number of EMR features may adversely affect performance and accuracy. To analyze EMR data with a large number of features and improve the assessment effectiveness, NMF was utilized to significantly reduce data dimension, discover latent factors, and improve data quality. Few studies have considered the application of NMF integrated with SVM [37] in patient phenotyping analysis. In the present study, a method integrating NMF with SVM is proposed to analyze diagnostic data for disease risk assessment.

To overcome the aforementioned limitations, an innovative approach is proposed for high precision RA prediction by using NMF. The main contributions of this study can be summarized as follows: 1) A novel framework called *eDRAM* (early disease risk assessment) is proposed for assessing disease risk in early development stages. In contrast with traditional practice, in the proposed framework, disease risk factors are approximately reconstructed by matrix factorization. 2) To the best the authors’ knowledge, this is the first study on matrix factorization techniques integrated with machine learning for disease risk assessment based on a nationwide medical diagnostic database. For a large number of diagnostic attributes, the proposed method can effectively approximate an optimal dimensionality. This improves both performance and data quality. 3) In the experiments, comprehensive evaluations were performed by comparing the proposed method with CBS, BayesFM, and CMAR for disease prediction. The results demonstrate that *eDRAM* is more effective than the other methods in terms of disease risk assessment metrics. To make the experiments more robust, wide-coverage data were used, and a sufficient number of evaluations were performed.

## Methods

### Overview of the proposed framework

**Fig 2:**
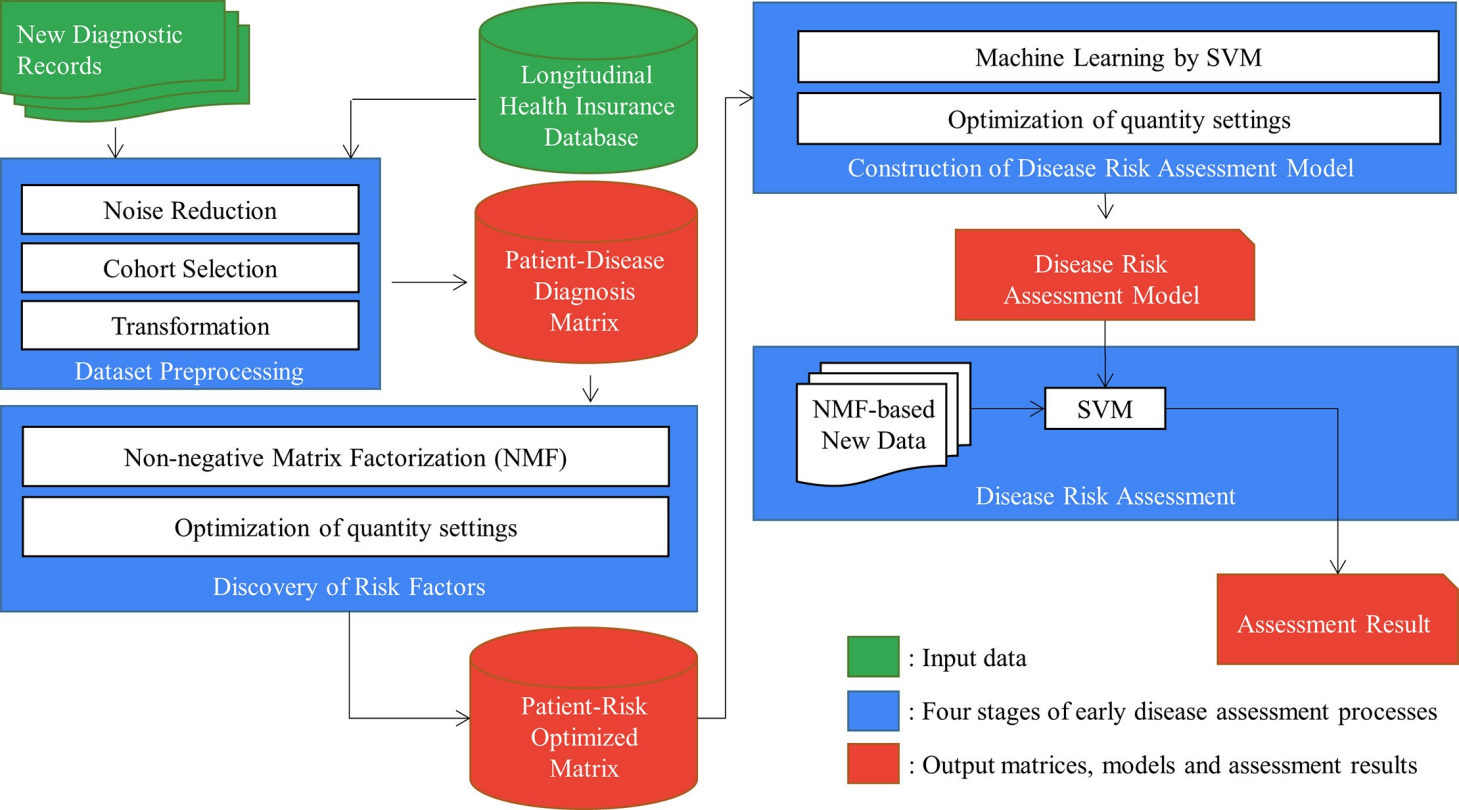
Framework of proposed approach.

Fig 2 shows the framework of the proposed *eDRAM* approach. It comprises three main stages: data preprocessing, risk factor optimization, and early disease risk assessment. The preprocessing stage comprises noise reduction, cohort selection, and matrix transformation. To discover the optimized risk factors, the NMF algorithm with parameter optimization was used for constructing the NMF-based matrix. In the assessment model learning and early disease risk assessment stages, the machine learning classifier SVM was used for disease modeling with the NMF-based matrix, yielding the final disease risk assessment, which serves as an excellent reference for physicians and patients. The instructions for executing the experimental protocols is available at dx.doi.org/10.17504/protocols.io.rv2d68e.

### Data preprocessing

#### Noise reduction

The EMR database contains noise, which may lead to biased disease risk assessment. Three types of data noise should be eliminated from the study cohort: 1) Incorrect data formats, such as inconsistent ICD-9-CM encoding rules or patient identification numbers with erroneous lengths. To determine this, the ICD-9-CM codes were formatted to five–digit codes. For instance, the formatting code 714.0, which represents RA, was formatted to 71400. 2) Missing, incomplete, or unreasonable data. 3) Meaningless or garbled data. For noise reduction, 795 records were removed.

#### Cohort selection

The patient data that were collected from the EMR database satisfied selection criteria related to the following: the time-period of the study, the ICD-9-CM codes of the studied disease, and the search strategy in the two subject databases.

**Fig 3:**
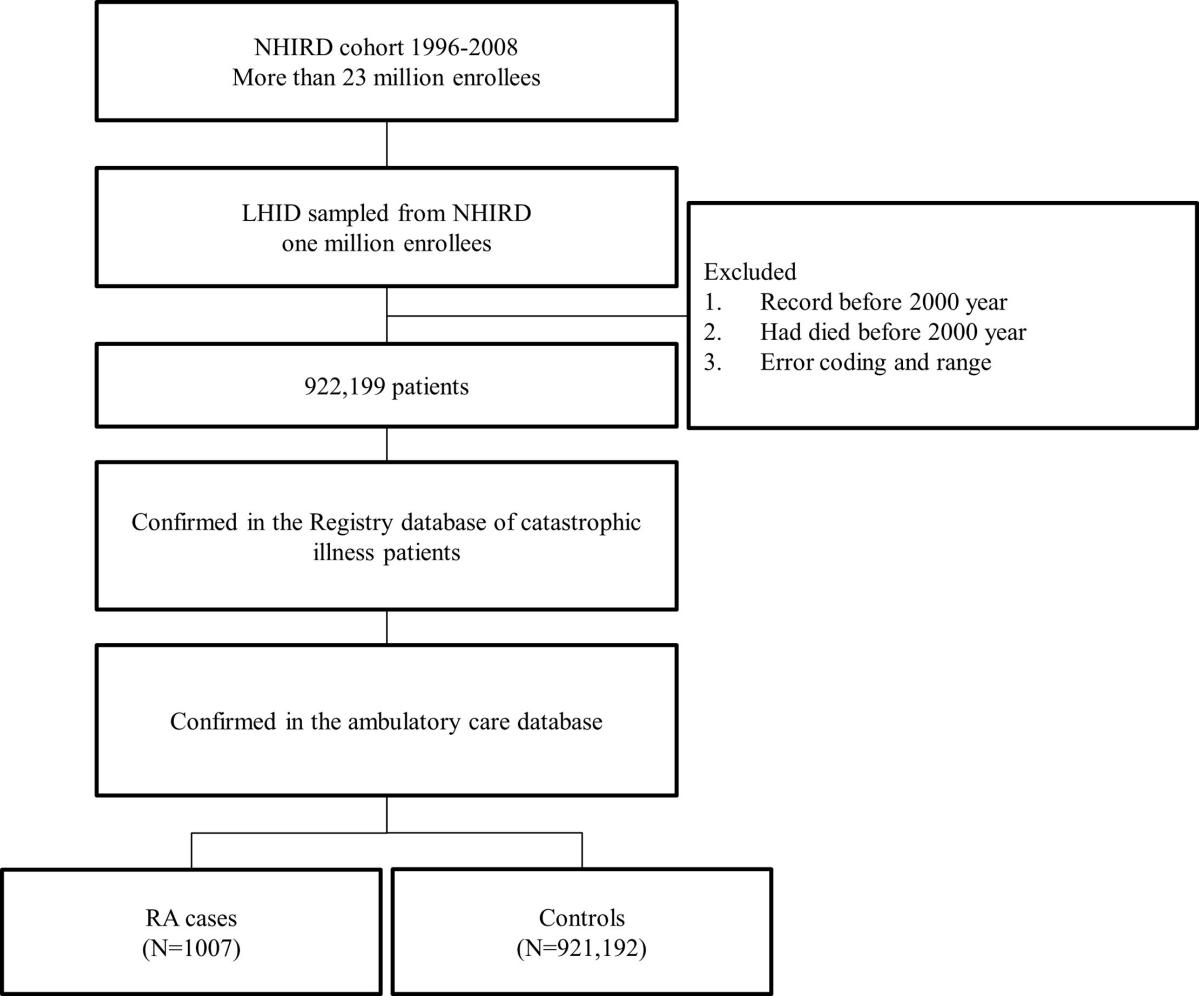
Flow chart of study cohort enrollment.

The original NHIRD contains information from 1996 to 2008; ICD-9-CM codes were adopted on January 1, 2000. To ensure consistent and standard codes, the cohort with outpatient diagnoses was used from 2000 to 2008. All RA cases met the criteria of the ICD-9-CM code 714.0 and were confirmed by using the registry of patients with catastrophic illnesses and the ambulatory care databases. RA cases were excluded in the controls. The cohort selection procedure flowchart is shown in Fig 3.

#### Data transformation

**Fig 4:**
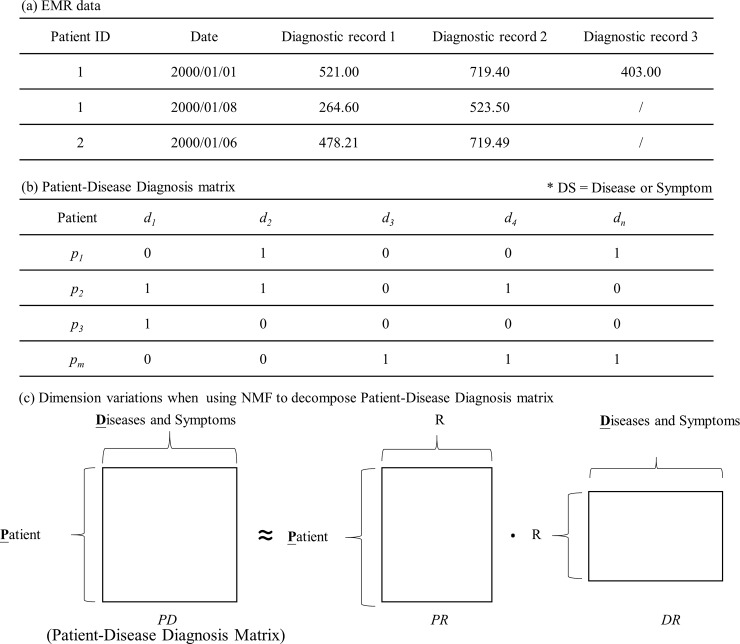
Example and concept of transformed patient–disease diagnosis matrix.

The outpatient clinical data analyzed here include patient ID, visiting date, and diagnostic disease codes generated at each clinic visit. An example is shown in Fig 4(A).

To analyze the relationship between the patient and the disease, the patient–disease diagnosis matrix is proposed by adopting a novel matrix-based analysis approach involving disease alignment for each patient. Given an *N* × *M* matrix, each row represents the medical history of a diagnosed patient across all diseases or symptoms (*DS*). Each column indicates the diagnostic record status of all patients for a single *DS*. The patient–disease diagnosis matrix is defined as follows.

**Definition 1** (*patient–disease diagnosis matrix*). Given a set of unique patients *P* = {*p*_1_, *p*_2_, …, *p*_*m*_, …, *p*_*|P|*_} (the total number of patients is |*P*|) and a set of unique diagnostic codes *D* = {*d*_1_, *d*_2_, …, *d*_*n*_, …, *d*_*|D|*_} (the total number of diagnostic codes in the EMR cohort is |*D*|), then the patient–disease diagnosis matrix is defined as *PDP→D* [*v*_*m*,*n*_], where *D* is the diagnostic code set, and *v* is a binary value (0 or 1), representing true or false for 0 *< n* ≤ |*D*|. Fig 4(B) shows an example of a patient–disease diagnosis matrix.

### Discovery of latent risk factors

After the patient–disease diagnosis matrix has been generated, the next operation is to approximate a better information matrix by executing the NMF algorithm.

NMF is a multivariate analysis algorithm for matrix factor optimization, matrix decomposition, and factor reconstruction [32–39]. It should be noted that the matrix model cannot contain negative values and is suitable for the analysis of medical diagnostic data after the transformation of the patient–disease diagnosis matrix. In this operation, the aim is to reduce the matrix dimension and to discover the latent risk factors. The new risk factors are multiplicative factors that are hidden among the original factor relationships, and their discovery allows more effective and efficient disease risk assessment. By Definition 1, the patient–disease diagnosis matrix is approximated by the two matrices in Eq (1). An example is shown in Fig 4(C).

$${PD}_{P\to D}[v_{n,m}]\approx {PR}_{P\to R}[pv_{n,r}]\cdot {DR}_{D\to R}{\left[dv_{m,r}\right]}^{T}.$$ (1)

*PR* and *DR* represent the factor matrices, each patient *p* and disease *d* are modeled by a factor vector set *R*, 0 < *r* ≦ |*R*|, and the elements in the two matrices are nonnegative.

The cost function, which quantifies the approximation, is defined as follows:

$${\left\|PD-PR∙{DR}^{T}\right\|}^{2}=\sum_{n,m}{\left(v-\sum_{r=1}^{\left|R\right|}pv_{n,r}-{dv}_{m,r}\right)}^{2}.$$ (2)

Eq (2) is minimized by multiplicative algorithms using Eq (3), which iteratively updates and improves the latent risk factors [32, 33, 35, 38, 45].

$$pv_{n,r}\leftarrow pv_{n,r}\frac{{\left(PD\cdot DR^{T}\right)}_{n,r}}{{\left(PR\cdot DR^{T}\cdot DR\right)}_{n,r}}\;\mathrm{and}\;v_{m,r}\leftarrow dv_{m,r}\frac{{\left(PR^{T}\cdot PD\right)}_{m,r}}{{\left(DR\cdot PR^{T}.PR\right)}_{m,r}}.$$ (3)

**Fig 5:**
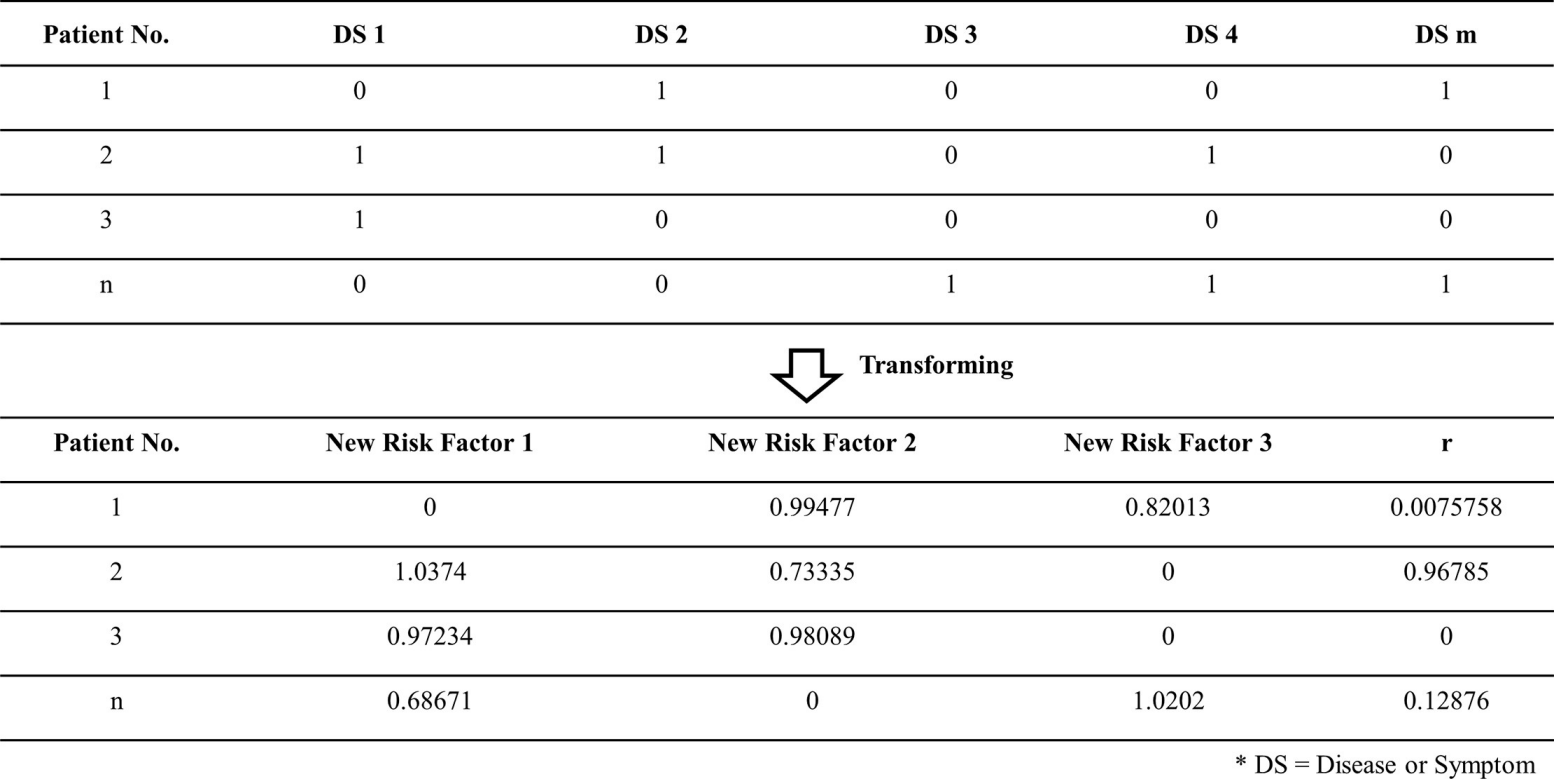
Using NMF to decompose the NMF-based matrix from the patient–disease diagnosis matrix.

According to the NMF algorithm, *PD* is decomposed into two risk factor matrices, namely, *PR* and *DR*. *PR* is called NMF-based matrix and contains the novel disease risk factors applied for disease risk assessment. For the example in Fig 4(B), the results of NMF are shown in Fig 5.

### Construction of disease risk assessment model

For constructing the disease risk model, SVM is applied to the stage for learning NMF-based matrix. This learned model can be a support to the disease risk assessment phase.

### Disease risk assessment

In this stage, the goal is to identify RA patients with disease risk from a cohort. Based on the disease risk assessment model, diagnostic records of unknown patient can be predicted using the SVM classifier [46]. Because SVM is a well-known classifier widely used by recent researches in the field of machine learning [9, 11, 18, 37, 44], it will not be described here further.

### Parameters

#### Parameters of non-negative matrix factorization

After the patient–disease diagnosis matrix transformation, NMF [33] was utilized for discovering risk factors by decomposing the patient–disease diagnosis matrix, yielding a new risk matrix of size *N* × *R*, where *R* must be less than *M* to reduce the factor dimensions and thus achieve data compression. As adjustments to *R* can affect the effectiveness of disease risk assessment, an optimal *R* value must be experimentally determined for each individual database.

#### SVM parameters with RBF kernel function

The SVM parameters are C and γ. C indicates the extent to which misclassification should be avoided, and thus higher values represent higher sensitivity. γ defines how far the influence of a training example reaches. Larger γ values form a small support vector, resulting in overfitting. The best combination of the two parameters can be obtained using the grid search method [46]. In this experiment, *C* was set to 2 and *γ* was set to 0.03125.

## Materials

In this study, a large-scale nationwide medical outpatient dataset, namely, Longitudinal Health Insurance Database 2000 (LHID2000) sampled from Taiwan’s NHIRD was used. NHIRD covers more than 99.6% of the general population of Taiwan, with approximately 23 million people [8], and is thus highly representative. The data is from the period 1996–2008.

To ensure that in the proposed approach, the specified disease model is appropriately learned, the database was divided into two datasets: RA cases and controls. The definitions of the two datasets are as follows: 1) RA cases included patients diagnosed more than twice with the RA diagnostic code and who were simultaneously enrolled in the registry database of patients with catastrophic illnesses. The RA patient data were collected from 2000 until the patients were diagnosed with RA for the first time (Fig 1). The hypothesis is that the disease patterns/models were hidden in the diagnostic records as early features/symptoms/relationships before the formal diagnosis of RA. The data after the patients were diagnosed with RA were not included in this dataset. The proposed method assesses whether patients would develop RA based on diagnostic data that were recorded before RA had been formally diagnosed. 2) Patients who did not meet the criteria that define RA and had medical diagnostic records from 2000 to 2008 were classified as controls.

In the cohort, there were a large number of outpatient diagnostic records of approximately 163 million individuals, containing 13,392 ICD-9-CM codes that also represented a number of diseases/symptoms. Each code represented a specific disease or symptom. With regard to gender, a statistically significant difference was observed, namely, the proportion of women in the dataset consisting of RA cases and controls was 76.3% and 48.9%, respectively, suggesting that the dataset consisting of RA cases had a larger number of women. The means of diagnostic records, diagnostic codes, and clinical meetings per year exhibited statistically significant differences in the comparison, indicating that RA cases involved more frequent meetings with physicians as well as more types of diseases. The frequency and distribution of the continuous variables for all patients were compared using Student’s *t*-test and Pearson’s chi-squared test. The prevalence of RA in the cohort is 0.1%, which is approximately equal to that reported in a previous study in Taiwan (97.5 cases in a population of 100,000) [47]. More details are shown in Table 1.

**Table 1:** Baseline characteristics of RA patients in the cohort (2000–2008).

	RA cases (N = 1007)	Controls (N = 921,192)	p-value
Mean age (SD), years	57.76 ± 15.41	41.78 ± 20.32	< 0.0001
Female, %	76.3	48.9	< 0.0001
Clinic visits			
No. of all visits	88,713	109,777,857	
Mean no. per year	21.59 ± 15.72	14.10 ± 12.6	< 0.0001
Median no. per year	18	10.57	
Diagnostic records			
No. of all diagnostic records	141,394	163,043,706	
Mean no. per year	34.51 ± 29.84	21.12 ± 22.80	< 0.0001
Median no. per year	26.31	13.87	
Diagnostic codes (ICD-9-CM)			
No. of diagnostic codes	2328	13,392	
Mean no. per year	7.98 ± 4.93	4.19 ± 2.56	< 0.0001
Median no. per year	6.96	3.7	

## Experiments

In this section, the details of the experiments are presented, namely, experimental dataset, experimental environment, experimental measures, experimental settings for parameter R, effectiveness evaluation, efficiency evaluation, and discussion.

### Experimental dataset

The experimental data were randomly sampled from the cohort (Table 1). They contained three sets, namely, Datasets 1, 2, and 3. Dataset 1 was used for the experimental setting of parameter R. Dataset 2 was used to evaluate performance. Dataset 3 was used to evaluate efficiency. As shown in Table 2, Dataset 1 consisted of 500 RA patients and 500 non-RA patients, Dataset 2 consisted of 500 RA patients and 25000 non-RA patients, and Dataset 3 consisted of 25000 RA patients and 25000 non-RA patients. The patients in Dataset 1 were different from those in Dataset 2. In Dataset 2, the non-RA patients were divided into 50 groups of controls, with 500 patients in each group. Each group had the same RA patients and different non-RA patients. Thus, Dataset 2 was divided into 50 new datasets. In Dataset 3, the number of RA patients was replicated from 1000 to 25000 and 25000 non-RA patients.

**Table 2:** Description of experimental data.

Description	Dataset 1	Dataset 2	Dataset 3
RA Patient No.	500	500	25000
non-RA Patient No.	500	25000	25000

**Fig 6:**
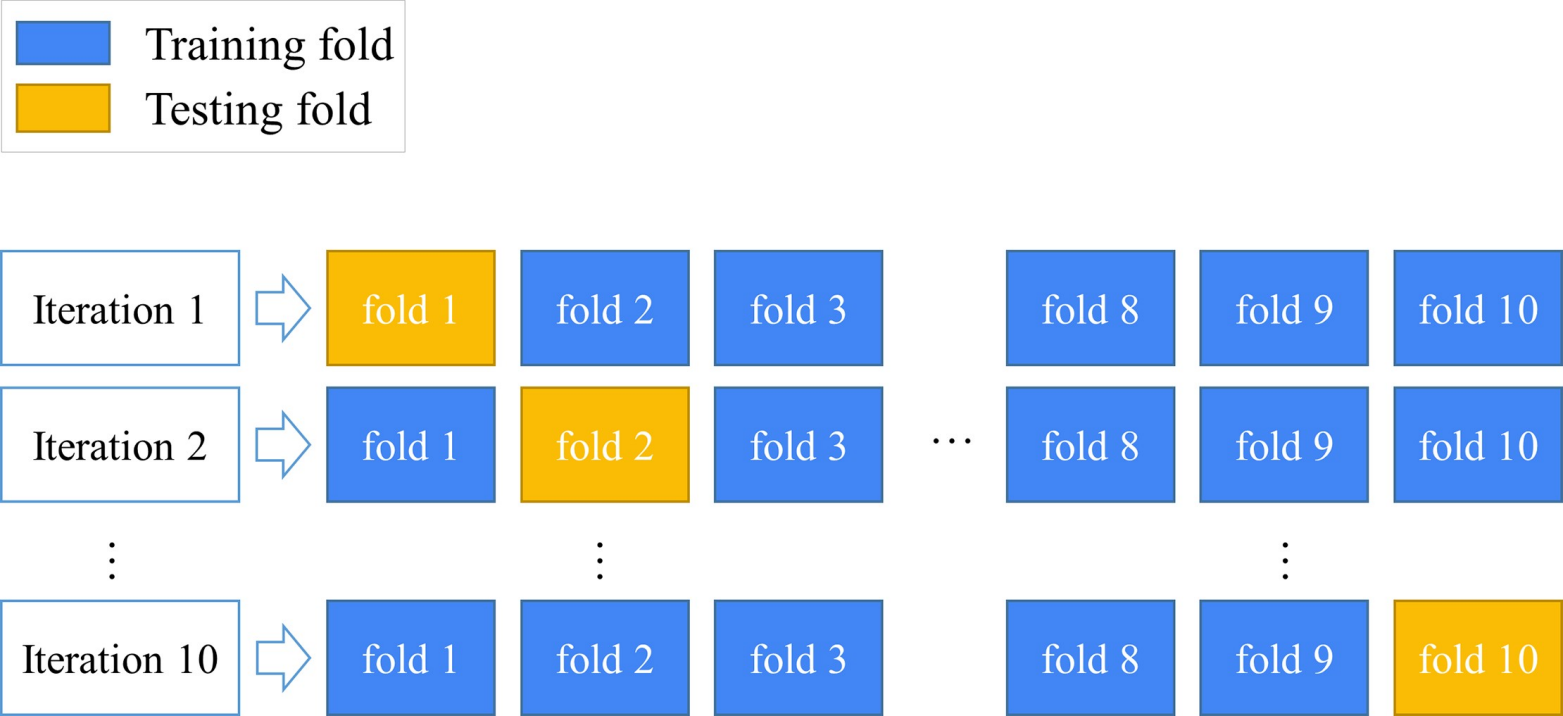
10-fold cross–validation model.

The dimension of each dataset was reduced by performing NMF separately. In the validation step, the stratified 10-fold cross-validation strategy [46, 48] was performed according to the proportion of the categories (RA and non-RA), with each fold having an equal proportion of RA patients and non-RA patients. Each iteration comprised nine folds as training data for construction of the disease risk model and one fold as testing data for performance evaluation. Ten iterations were performed in sequence. The results were averagely calculated (Fig 6).

### Experimental environment

The experiments were implemented on a server with two Intel CPU E5-2630 v4 2.2GHz and 32GB RAM, running Windows 7 Enterprise. All classification algorithms were implemented in Java. The NMF library of Matlab 2016a and libSVM [46] were used in the study.

### Experimental measures

To evaluate the proposed method, the following metrics were employed: accuracy, sensitivity, specificity, and standard deviation. They are described as follows, and the corresponding formulas are given in Eqs 4–7. *True positive* is the number of RA cases correctly assessed. *True negative* is the number of control cases correctly assessed. *Condition positive* is the number of all RA cases. *Condition negative* is the number of all control cases. *Sensitivity* (equivalent to recall) represents the ability to correctly assess patients with RA. *Specificity* indicates the ability to correctly assess patients without RA. *Accuracy* indicates the ability to correctly assess the cases of RA and controls. In the experiment setting phase, the purpose of adjusting the parameters is to balance sensitivity and specificity with the highest performance. Accuracy can be regarded as the average of sensitivity and specificity, as the proportion of patients has been adjusted. Standard deviation (SD) is used to measure the amount of variation of a set of measurements (sensitivity, specificity, and accuracy). This represents a measure of stability of a classifier. If {*x*_1_, *x*_2_, …, *x*_*n*_} are the observed values, *μ* is their mean value, and *n* is their number, then

$$Accuracy=\frac{\sum True\;positive+\sum True\;negative}{\sum Condition\;positive+\sum Condition\;negative},$$ (4)

$$Sensitivity=\frac{\sum True\;positive}{\sum Condition\;positive},$$ (5)

$$Specificity=\frac{\sum True\;negative}{\sum Condition\;negative},$$ (6)

$$Standard\;Deviation=\sqrt{\frac{1}{N}\sum_{i=1}^{N}{\left(x_{i}-\mu \right)}^{2}}.$$ (7)

These metrics were used to compare the proposed *eDRAM* method with three representative approaches, namely, *CBS* [29], *CMAR* [27], *and BayesFM* [31]. To obtain a highly effective disease risk assessment, the parameters in this experiment should be adjusted using all approaches. The details are described below.

### Experimental results

In the experiments, the evaluations comprised: 1) Selection of the R value based on effectiveness. 2) Effectiveness comparisons between the proposed method and existing well-known disease risk assessment systems in terms of sensitivity, specificity, accuracy, and standard deviation of specificity. 3) Efficiency evaluation of all methods.

#### Experimental settings for parameter R

For NMF, the diagnostic data should be transformed to the patient-disease diagnosis matrix. The number of diseases/symptoms used in the patient-disease diagnosis matrix was 13392. To optimize the NMF-based risk factors, the *R* value should be determined. It represents the number of risk factors refined from the *M* columns of the original disease diagnostics matrix, where *R* < *M*. Particularly, overly large or small values of *R* would decrease the effectiveness of disease risk assessment. To determine the optimal *R* value, experiments were conducted by using Dataset 1 and varying *R* from 100 to 900 with an interval of 100.

**Fig 7:**
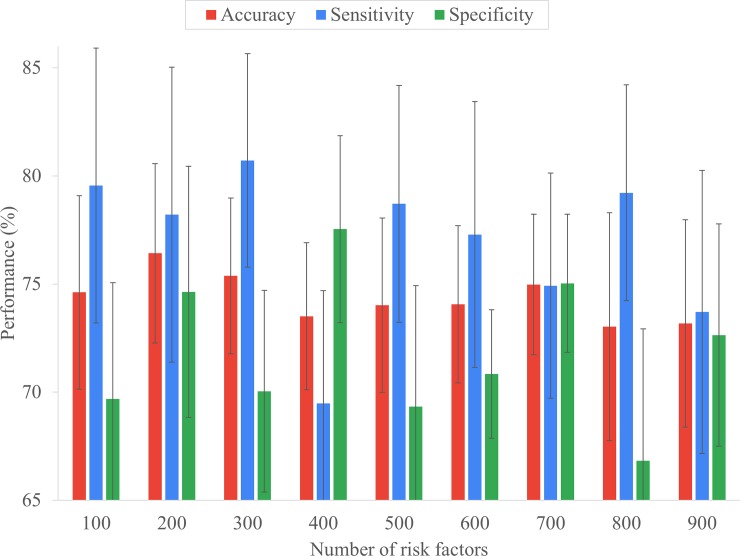
Effectiveness of RA risk assessment under different number of risk factors.

Fig 7 shows the effectiveness of RA assessment for various risk factor numbers. The following should be noted. First, the trend of the curve shows that high *R* values result in relatively low accuracy. When the number of risk factors reaches 800, an unstable assessment is obtained, that is, the difference in sensitivity and specificity is greater than 10%. This implies that excessive risk factors will reduce accuracy. Secondly, as the *R* value decreases, the measured value tends to stabilize and converge. However, lower *R* values will reduce the overall measurement values resulting in observations varying from 200 to 100. Thirdly, the measure values has a cyclic relationship with the trend of the *R* value. For example, in Fig 7, when the *R* value is in the range 400–700, the continuous value of the sensitivity forms a peak. Fourthly, accuracy can be considered a combination of sensitivity and specificity. Fifthly, when *R* is 200, the accuracy and its standard deviation achieved the best result. Based on the highest accuracy (the lowest standard deviation and the acceptable distance between sensitivity and specificity was smaller than 5%), *R* was set to 200 for the following experiments.

#### Effectiveness evaluation

**Fig 8:**
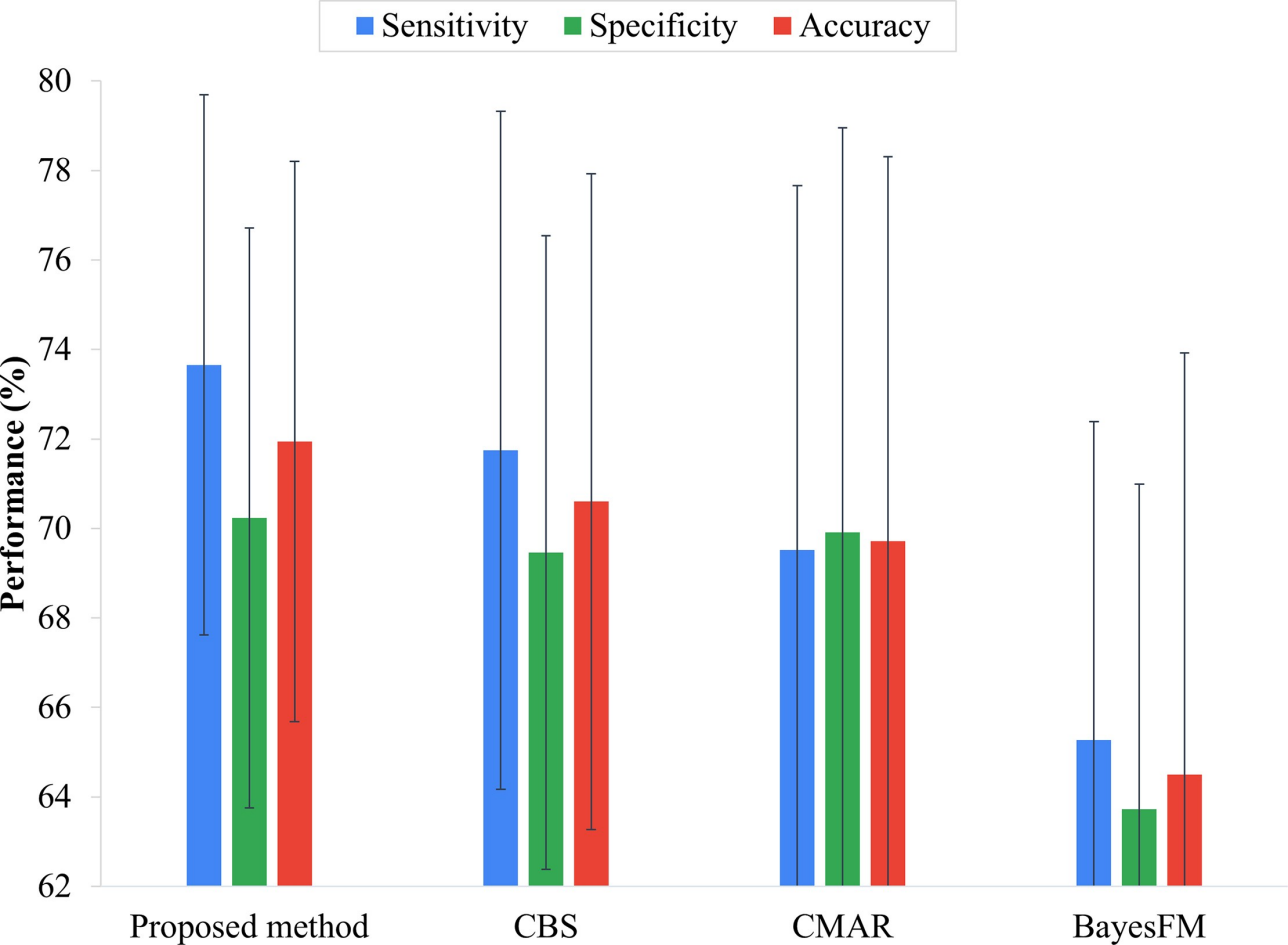
Comparison of the performance of the *proposed method*, CBS, CMAR, and BayesFM approaches.

In this experiment, the performance of the proposed framework *eDRAM* was evaluated against CBS [29], CMAR [27], and BayesFM [31] on Dataset 2. The min_sup values of CBS, CMAR, and BayesFM were set to 0.063, 0.005, and 0.006, respectively. The comparison shows that *eDRAM* achieved a better assessment rate than CBS, CMAR, and BayesFM in terms accuracy, sensitivity, and specificity on the cohort (Fig 8). Moreover, *eDRAM* maintained sensitivity and specificity closer to each other and better balanced than the other approaches (Fig 8); thus, *eDRAM* is more practical. Furthermore, *eDRAM* proved highly efficient, as it used fewer risk factors and still achieved better efficacy. Indeed, the number of risk factors in *eDRAM* was reduced to 200 (Fig 7), whereas the other approaches had 13,392 risk factors (Table 1).

The stability of the proposed disease risk assessment approaches is now evaluated. The experiment was based on the results of the previous sub-section for the standard deviation of sensitivity, specificity, and accuracy. The standard deviation was used (Eq 10) to evaluate the performance stability of the approaches. When the standard deviation is smaller, stability is higher. Fig 8 shows the results of comparing *eDRAM*, CBS, CMAR, and BayesFM in terms of the standard deviation of sensitivity, specificity, and accuracy. It can be seen that *eDRAM* is the most stable method because it converts visible variables to latent factors [32, 33] that are non-redundant and more concise so as to achieve high-quality disease risk assessment.

#### Efficiency evaluation

In this experiment, the average runtime in the assessment phase was calculated by using Dataset 3. The experimental results in Table 3 show that the proposed method had the best performance in terms of assessment time. In the assessment phase, the proposed method was 2.5 times as efficient as CMAR and five times as efficient as CBS. Owing to dimensionality reduction, the proposed method can reduce loading and execution time. Regarding the other methods, the diagnostic datasets have several features that result in an increase in the number of disease patterns and hence an increase in the search time during the disease risk assessment phase.

**Table 3:** Efficiency comparison on Dataset 3.

	Proposed method	CMAR	CBS	BayesFM
Assessment time (sec.)	0.12	0.30	0.61	41.90

## Discussion

Based on the performance and the stability measures obtained from the experimental evaluation, the following can be concluded:

1. The experimental evaluation demonstrates that *eDRAM* is superior in terms of accuracy, sensitivity, and specificity (Fig 8), as it establishes a matrix-based diagnostic data analysis model to decompose the NMF-based matrix for identifying important disease risk factors. This indicates that the proposed approach has the advantage of finding more associated factors hidden in the diagnostic data than the other approaches.
2. Fig 7 shows the trade-off between sensitivity and specificity, that is, it is not easy to perform well in terms of both sensitivity and specificity. For example, Fig 8 shows CMAR has better specificity than CBS but is poor in terms of sensitivity. By contrast, with *eDRAM*, both sensitivity and specificity are robust for early disease risk assessment.
3. In disease risk assessment, stability and efficacy are equally important. Therefore, an experiment involving standard deviation was conducted to evaluate stability. It was demonstrated that high performance can be achieved, but its stability is not necessarily optimal. For example, Fig 8 shows that CBS ranks second in performance, but third in stability. The experimental evaluation shows that *eDRAM* is reliable in terms of both stability and performance for early disease risk assessment.
4. In the experiment for selecting *R*, the dimension was reduced from the original 13392 to 200, the ratio being approximately 66. Even though the amount of data was significantly reduced, the experimental results on performance demonstrated that the proposed method achieved better results compared to the other methods.
5. In the experiment, BayesFM discovered an excessively large number of sequential rules (features). Although the pruning algorithm was employed, there was still a large number of rules employed for assessment (> 25000), which is 50 times more than in CBS. This indicates that an excessively large number of features will result in low efficiency (Table 3), lower effectiveness (Fig 8), and render the assessment results unstable (higher standard deviations, as shown in Fig 8).
6. The patient-disease diagnosis matrix is transformed into a NMF-based matrix with significantly reduced dimensions, instead of selecting specific factors. The NMF-based matrix is still associated with the original matrix and can approximate it. For machine learning, an overly large number of attributes and less data correlation may lead to misjudgment and reduce efficiency. Thus, NMF is suitable for extensive analysis of big data [34, 49, 50].

## Conclusions

Several serious diseases are not apparent during the early stages of their development. Hence, they are difficult to diagnose. This delayed detection results in missing the optimal time for treatment initiation that may be critical for controlling the disease. To address this, a novel method called *eDRAM* was proposed for early disease risk assessment with high efficacy, efficiency, and stability. *eDRAM* discovered novel risk factors from a large-scale nationwide outpatient diagnostic database using matrix factorization. Based on the optimal risk factors discovered, a disease risk assessment model was established using machine learning techniques. Thereupon, the model successfully assessed the disease risk. In summary, the contributions of this study are as follows. First, to the best of the authors’ knowledge, this is the first study to apply the NMF algorithm. The main advantages of the proposed method using NMF lie in that the optimal factors hidden in the data can be approximated to achieve high assessment accuracy, and the traditional problems of big data can be resolved by significant dimensionality reduction. Secondly, a diagnostic data model called patient-disease diagnosis matrix was proposed for mapping the medical diagnostic dataset. It facilitates further data analysis and factor discovery by using matrix factorization and classification techniques, as was demonstrated in this study. Moreover, it provides a new perspective for the problem of disease risk assessment. Thirdly, modeling of disease risk assessment based on the longitudinal nationwide EMR is effective, reliable, and robust. The experimental results demonstrated that the proposed approach is superior to the three modern classification approaches used for disease risk assessment.

For future work, several research directions could be further explored. First, medications play an important role in disease treatment. Hence, the prescription database and associations between prescriptions and diseases can be considered important risk factors. Secondly, environmental conditions associated with diseases, such as place of residence, season, and occupation, are potential risk factors that should be taken into consideration to enhance the effectiveness of disease risk assessment. Thirdly, as temporal information is an important potential factor, a temporal information model could be advantageously incorporated. Finally, using the proposed matrix-based analytic approach in combination with novel effective classifiers can aid in the discovery of deeper risk factors and the early detection of several serious diseases.
